# Development of a Monoclonal Antibody-Based Indirect Competitive Enzyme-Linked Immunosorbent Assay for the Rapid Detection of Gallic Acid

**DOI:** 10.3390/bios14040182

**Published:** 2024-04-09

**Authors:** Jiajing Duan, Xiuxia Zheng, Ran Tao, Long Li, Fengzhong Wang, Yufeng Sun, Bei Fan

**Affiliations:** Institute of Food Science and Technology, Chinese Academy of Agricultural Sciences, Beijing 100193, China; duanjiajing2021@163.com (J.D.); 18646654981@163.com (X.Z.); ran.tao5@mail.mcgill.ca (R.T.); llzgnydx@163.com (L.L.); wangfengzhong@sina.com (F.W.); fanbei@caas.cn (B.F.)

**Keywords:** gallic acid, monoclonal antibody, ic-ELISA, hapten, rapid detection

## Abstract

Gallic acid (GA) is closely related to the quality of herbal medicines and other agricultural products. In order to facilitate the rapid detection of GA, we developed a monoclonal antibody-based ic-ELISA method. Antigens with and without connecting arms were prepared. It was found that the introduction of connecting arms (linear carbon chain) was beneficial for immune response. By utilizing hybridoma technology, a specific mAb (anti-GA-M702) was screened and identified, which exhibited a 1:40,500 antibody titer and IgG2b antibody subtype. The ic-ELISA assay was established based on anti-GA-M702. The optimal working concentrations of the encapsulated antigen and antibody were 0.5 µg/mL and 0.67 µg/mL, respectively. The ic-ELISA method showed a linear detection range of 297.17–2426.61 ng/mL for GA with a sensitivity of 849.18 ng/mL. It displayed a good applicability for the determination of GA in *Galla chinensis*. In conclusion, the ic-ELISA method provides an efficient approach to the rapid detection of GA in products.

## 1. Introduction

Gallic acid (GA) is a polyphenolic compound existing in many plants, such as Chinese rhubarb (*Rheum officinale* Baill.), Blue Gum (*Eucalyptus globules* Labill), and Medical Dogwood (*Cornus officinalis* Sieb. et Zucc.). It is valued for its antioxidant [1], anti-inflammatory [2], and antimicrobial [3] activities. GA is widely recognized as the characteristic and major effective component of multiple Chinese herbal medicines, such as *Schisandra chinensis*, *onychospermum officinale*, and *Eugenia ciliata*. It is also recognized as a key taste-active compound of some wine, tea, and fruit [4,5]. Ng et al. found that the content of GA in 12-, 17-, and 30-year Scottish wines was 2.4, 7.0, and 37.6 mg/L, respectively, showing the trend that the content of GA in Scottish wine significantly raised with the increase in vintage [6]. Li et al. found that GA negatively affected the sweet-mellow sensation of congou black tea infusions [7]. GA is also considered to be related to the maturity of some fruits, such as mango, banana, and persimmon [8]. Therefore, establishing efficient techniques for determining GA can provide support for the quality assessment and quality control of various agricultural products.

A number of detection methods for GA have been developed, mainly including high-performance liquid chromatography (HPLC) [9], gas chromatography (GC) [10], differential pulse polarography (DNP) [4], and electrochemistry (EC) [11]. The detection methods for GA are summarized in Table 1. Despite being sensitive, some shortcomings (e.g., costly equipment and trained personnel) make the methods unable to be used on-site. In contrast, immunoassay methods are time-saving, convenient, and cost-effective. They are extensively used for quality control in food industries [12] and environmental pollutant analysis [11]. However, so far, few immunoassay methods have been developed for the detection of GA.

In this study, we designed and synthesized antigens with and without connecting arms and prepared a monoclonal antibody (mAb). Based on this mAb, we established an indirect competitive enzyme-linked immunosorbent assay (ic-ELISA) for detecting GA.

## 2. Materials and Methods

### 2.1. Reagents and Instruments

GA, N-(3-bromopropyl) benzene diamine, butyric acid, protocatechuic acid, benzoic acid, caffeic acid, ferulic acid, eugenol, and gallicin were obtained from Macklin (Shanghai, China). N-Hydroxy succinimide (NHS) and 1-(3-Dimethylaminopropyl)-3-ethylcarbodiimide hydrochloride (EDC) were purchased from Yuanye (Shanghai, China). K_2_CO_3_, ethyl acetate, dimethylformamide (DMF), bovine serum albumin (BSA), and ovalbumin (OVA) were purchased from SIGMA. Ten 8-week SPF male mice were obtained from Vital River Laboratory Animal Technology (Beijing, China), license number SCXK (Beijing): 2021-0006; SP2/0 myeloma cells were purchased from Cell Center, Institute of Basic Medical Sciences, Chinese Academy of Medical Sciences.

HPLC was performed with 1220 infinityIILC (Agilent, Santa Clara, CA, USA). High-resolution mass spectrometry (HRMS) was carried out using Q Exactive Focus (Thermo Fisher, Waltham, MA, USA). UV spectrophotometer was recorded with UV-8000S (Metash, Shanghai, China). MALDI-TOF was recorded with Bruker Autoflex III (Bruker-Spectrospin AG, Karlsruhe, Germany).

### 2.2. Synthesis of Hapten

**Figure 1:**
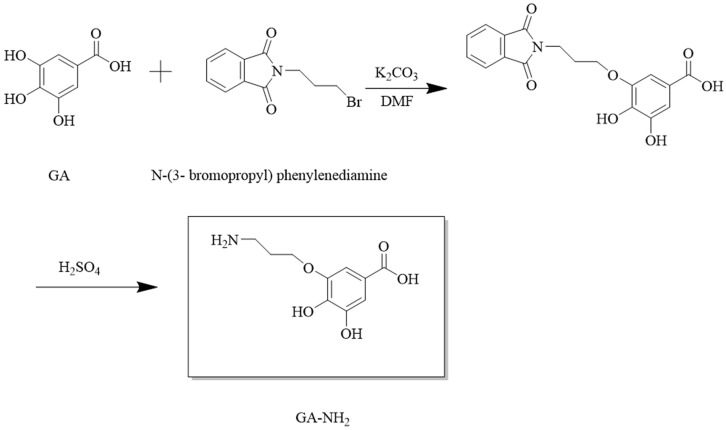
Synthetic route of hapten GA-NH_2_.

A novel hapten with GA moiety and a linear carbon chain was designed. It was synthesized according to the route shown in Figure 1. Briefly, GA (1.37 g), N-(3-bromopropyl) phenylenediamine (1.115 g), and K_2_CO_3_ (1 g) were dissolved in DMF (35 mL) and reacted at 40 °C for 2 h. The solution was extracted three times with ethyl acetate (100 mL) and washed three times with water (200 mL). The organic phase was added with water (50 mL) and 98% H_2_SO_4_ (25 mL). Then, hydrolysis reaction was carried on under acidic conditions at 25 °C for 3 h. The resulting solution was recrystallized to obtain hapten (GA-NH_2_). The hapten was characterized by HPLC and HRMS.

### 2.3. Preparation of Antigen

**Figure 2:**
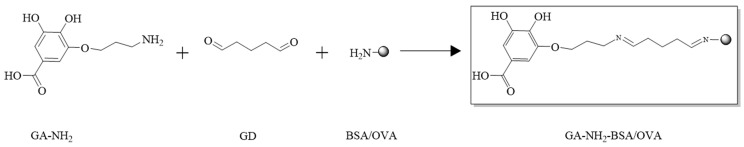
Synthetic route of immunogen GA-NH2-BSA and coating antigen GA-NH2-OVA.

Immunogen GA hapten BSA (GA-NH_2_-BSA) and coating antigen GA hapten ovalbumin (GA-NH_2_-OVA) were produced by coupling GA-NH_2_ with BSA and OVA according to the synthesis route shown in Figure 2. Briefly, GA-NH_2_ (4.7 mg) was dissolved in PBS (pH 7.4, 0.01 M, 2 mL), followed by the addition of glutaraldehyde (GD, 50%, 50 μL), and then reacted at 25 °C (controlled by thermostat) for 4 h. Then, the system was mixed with BSA solution (40 mg BSA dissolved in 4 mL of 0.01 M PBS) dropwise and reacted at 25 °C for 24 h. The resulting solution was dialyzed in PBS (pH 7.4) at 4 °C for 72 h to isolate GA-NH_2_-BSA (16.31 mg). The same method was used to prepare GA-NH_2_-OVA (17.55 mg). Both GA-NH_2_-BSA and GA-NH_2_-OVA were identified by MALDI-TOF.

**Figure 3:**
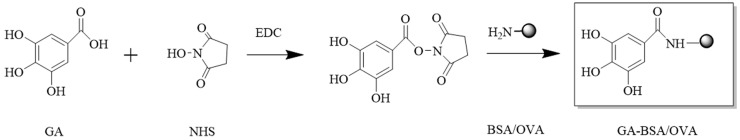
Synthetic route of immunogen (GA-BSA) and coating antigen (GA-OVA).

To compare the influence of the carbon chain length, the immunogen (GA-BSA) and coating antigen (GA-OVA) without carbon chain were prepared via an active ester method [19]. The synthesis route is shown in Figure 3. The dose of GA, BSA, and OVA were 4.7, 40, and 40 mg, respectively. The yields of GA-BSA and GA-OVA were 15.56 and 16.08 mg, respectively.

### 2.4. Preparation of mAb

The mAb was prepared according to previous research with some modifications [20]. Briefly, the immunogens GA-NH_2_-BSA (100 μg) and GA-BSA (100 μg) were emulsified with Freund’s complete and incomplete adjuvants, respectively. Ten BALB/c female mice (6–8 weeks of age) were fed for 2 weeks. They were randomly divided into two groups and immunized on the neck and back with GA-NH_2_-BSA and GA-BSA emulsified with Freund’s complete adjuvant, respectively. Then, the mice were immunized 5 times with the immunogens emulsified with Freund’s incomplete adjuvant, with an interval of 2 weeks between each immunization. After each immunization, the serum titer was tested. The mouse with the highest serum titer was chosen for intraperitoneal injection with the immunogens (50 μg) dissolved in normal saline (50 μL). Polyethylene glycol method was used for fusion of mouse spleen cells with SP2/0 cells. The positive cell lines were selected and injected into the abdomen of BALB/c female mice to obtain ascites. The mAb was obtained by the purification of ascites. The concentration of mAb was measured by a nanodrop ultra micro spectrophotometer. The mAb titer was determined by ELISA. And the mAb subtype was detected using the Antibody Type Detection Kit.

### 2.5. Establishment of ic-ELISA Analysis

#### 2.5.1. Determination of Optimum Concentration of Coating Antigen

The coating antigen was diluted with a carbonate buffer solution (0.05 M, pH 9.6) to different concentrations (1, 0.5, 0.25, 0.125, 0.0625, 0.03125 μg/mL). To each well of a 96-well plate, the coating antigen (100 μL) was added and incubated for 2 h at 37 °C. The plate was washed 3 times with PBS solution (0.01 M, containing 0.05% Tween 20, pH 7.4) and blocked with gelatin solution (100 µL, 10 mg/mL) as a blocking buffer, followed by incubation for 2 h at 37 °C and a washing procedure. mAb (100 µL, 2 µg/mL) was added to each well and incubated for 1 h at 37 °C, followed by another washing. GaMIgG-HRP (100 µL, diluted 10,000 times with phosphate-buffered saline) was added to each well and incubated for 1 h at 37 °C, followed by washing. Then, a 3,3′,5,5′-Tetramethylbenzidine (TMB) chromogenic solution (100 μL/well) was added and incubated for 0.5 h, followed by the addition of a stopping solution (50 μL/well). The absorbance was measured at 450 nm.

#### 2.5.2. Determination of Optimum Concentration of mAb

To each well of a 96-well plate, the optimum concentration of the coating antigen (100 µL) was added and incubated for 2 h at 37 °C. The plate was washed 3 times and blocked with a blocking buffer (100 µL, 10 mg/mL), followed by incubation for 2 h at 37 °C and washing procedure. A standard working solution of GA with different concentrations was added to the first row (50 µL/well). The operation was repeated for the rest rows. Varying concentrations of mAb (2, 0.67, 0.22, 0.074, 0.025 μg/mL) were added to the first column (50 µL/well). The operation was repeated for the rest columns. The plate was stored at 37 °C for 1 h and washed. The relevant steps for adding GaMIgG-HRP, TMB chromogenic solution, and stopping solution were the same as in 2.5.1. The absorbance was measured at 450 nm.

#### 2.5.3. Development of ic-ELISA Standard Curve

The concentrations of coating antigen and mAb were set to the determined optimum concentrations. The standard working solution of GA with different concentrations was set to 250, 500, 1000, 2000, 4000 ng/mL. The relevant steps for the envelope, block, and addition of different concentrations of the GA standard working solution, mAb, GaMIgG-HRP, TMB chromogenic solution, and stopping solution were the same as reported in Section 2.5.2. The absorbance was measured at 450 nm. The competitive inhibition curve for a quantitative analysis of GA was established, and the optimal detection range for GA could be calculated with the concentration of the analyte providing a 20–80% inhibition rate (IC_20_–IC_80_ values) [13]. The detection sensitivity could be calculated with the concentration of the analyte providing a 50% inhibition rate (IC_50_) [21]. Based on the established ic-ELISA standard curve, the GA analogues (syringic acid, protocatechuic acid, benzoic acid, caffeic acid, ferulic acid, and eugenol) were used to determine the cross-reaction rates.

### 2.6. Analysis of Galla Chinensis by ic-ELISA

Chinese herbal medicine, *G. chinensis*, was used to assess the applicability of the ic-ELISA analysis. Briefly, *G. chinensis sample* was determined with the concentration of 310.21 mg/g by HPLC. The powder (60 mesh) of the sample was eatracted by 80% ethanol with a material–liquid ratio of 1:10 (g/mL). The extraction solution was diluted 2 × 10^4^, 5 × 10^4^, 10^5^ multiples with PBS solution to ensure the concentrations were within the detection range. Then, the obtaining solutions were tested by ic-ELISA assay and the absorbance was measured at 450 nm. Each group was set with 3 parallels.

## 3. Results and Discussion

### 3.1. Synthesis and Characterization of Hapten and Antigen

**Figure 4:**
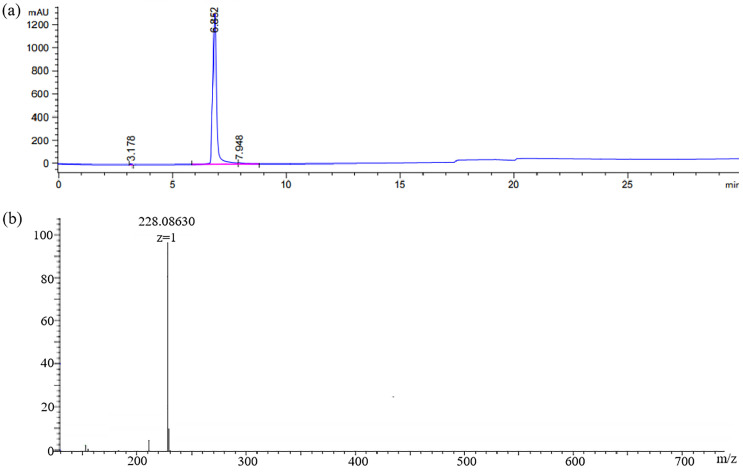
(**a**) HPLC chromatogram and (**b**) HRMS spectra of hapten GA-NH_2_.

In this work, the hapten GA-NH_2_ was acquired by introducing a linear carbon chain to the GA structure. Its purity (95%) was determined by HPLC (Figure 4a). HRMS (Figure 4b) showed [M + H]^+^: 228.08665 (theoretical value) and 228.08630 (measured value).

**Figure 5:**
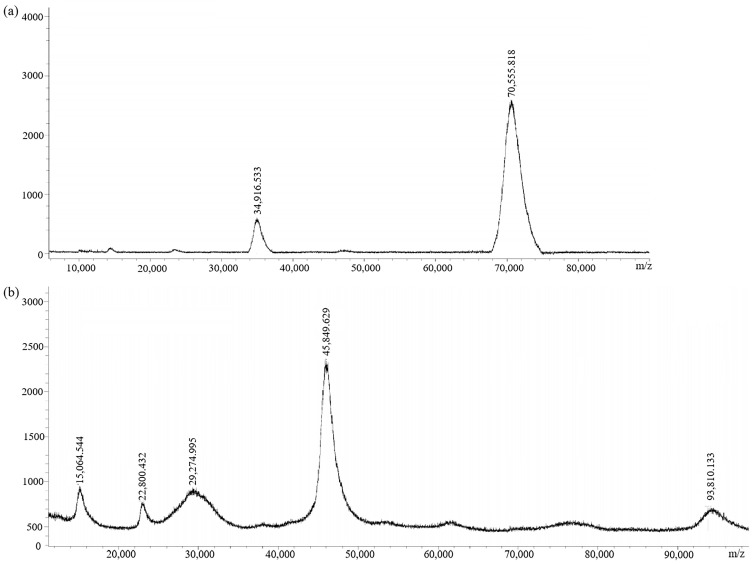
MALDI-TOF spectra of (**a**) immunogen GA-NH_2_-BSA and (**b**) coating antigen GA-NH_2_-OVA.

The immunogen (GA-NH_2_-BSA) and the coating antigen (GA-NH_2_-OVA) were prepared by conjugating GA-NH_2_ with BSA and OVA, respectively. MALDI-TOF (Figure 5) showed the molecular weights: 70,555.818 (GA-NH_2_-BSA) and 45,849.629 (GA-NH_2_-OVA). The coupling ratios of GA-NH_2_-BSA and GA-NH_2_-OVA were 15.2:1 and 3.4:1, respectively. The result indicated that GA-NH_2_-BSA could be used as an immune antigen and GA-NH_2_-OVA could be used as an encapsulated antigen.

**Figure 6:**
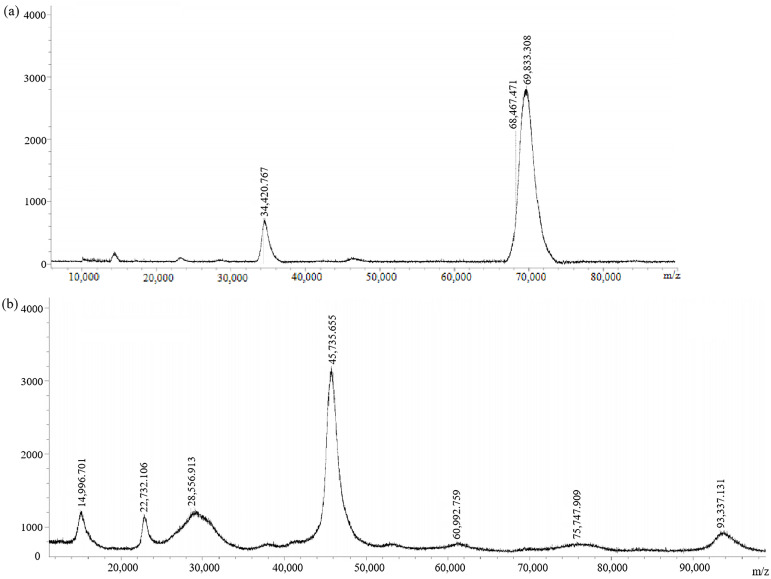
MALDI-TOF spectra of (**a**) immunogen GA-BSA and (**b**) coating antigen GA-OVA.

The antigens (immunogen GA-BSA and coating antigen GA-OVA) without connecting arms were also synthesized. They were also characterized by MALDI-TOF (Figure 6). The molecular weights of GA-BSA and GA-OVA were 69,833.308 and 45,735.655, respectively. GA-BSA and GA-OVA had a coupling ratio of 16:1 and 3.8:1, respectively. They were equivalent to GA-NH_2_-BSA and GA-NH_2_-OVA, respectively.

### 3.2. Production and Characterization of mAb

After immunizing the mice with GA-NH_2_-BSA and GA-BSA as immunogens, respectively, the serum titer was tested by routine ELISA procedures, respectively. The serum titer was represented by the dilution factor when the P/N value was greater than 2.1, where P was the A_450_ value of the immunized mice serum and n was the A_450_ value of the negative control. After boosting with GA-NH_2_-BSA five times, the serum titer of mice MSZ 1-1, MSZ 1-2, and MSZ 1-3 reached more than 27,000 (Table 2). The result indicated that GA-NH_2_-BSA successfully immunized mice and could be used for cell fusion experiments. While after boosting with GA-BSA five times, the serum titers of five mice were less than 1000 (Table 3), indicating that GA-BSA was not sufficient to produce an immune response in the mice. Therefore, GA-BSA could not be used for cell fusion experiments. Compared to the immune results of GA-NH_2_-BSA and GA-NH_2_-BSA, it was found that the introduction of connecting arms was beneficial for the exposure of antigenic determinants and the production of immune response.

The most commonly used cell fusion methods include physical methods (e.g., electrofusion and laser fusion), chemical methods (e.g., polyethylene glycol as fusion agent), and biological fusion methods (e.g., inactivation of Sendai virus). In this study, the chemical method with polyethylene glycol as a cell fusion agent was used. The splenocyte of mouse MSZ1-2 which had a high serum titer was selected for cell fusion with myeloma cell. The hybridoma cell line M702, which could stably secrete antibody, was obtained after several cell rounds of subcloning. The monoclonal antibodies produced by this cell line were named anti-GA-M702. Their concentration was 5.1 mg/mL and its titer was 1:40,500 (Table S1). The antibody subtype was determined to be IgG2b (Table S2).

### 3.3. Development of ic-ELISA Analysis

**Figure 7:**
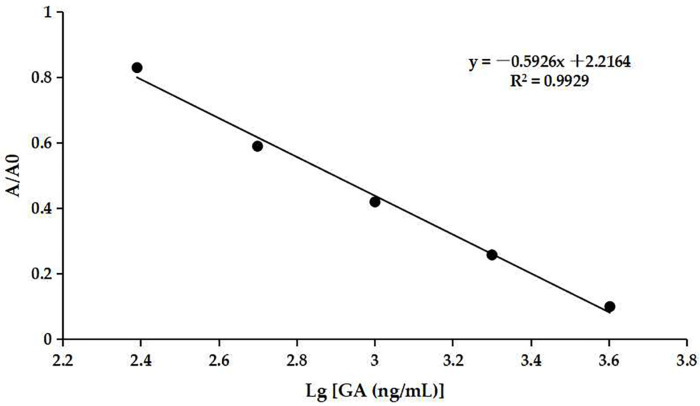
The ic-ELISA standard curve.

The optimum concentrations of coating antigen and mAb were determined to be 0.5 µg/mL (Table S3) and 0.67 µg/mL (Table S4), respectively. The ic-ELISA standard curve was established as shown in Figure 7. The assay showed an IC_50_ value of 849.18 ng/mL for GA and a detection range of 297.17–2426.61 ng/mL. The IC_20_ value was defined as the calculated limit of detection (cLOD), which was 297.17 ng/mL. The cLOD of the method was lower than some other methods, e.g., SPE-HPLC [16] and CE [18]. The result indicated that the developed ic-ELISA assay had good sensitivity to GA.

In general, a monoclonal antibody with high specificity has low cross-reactivity to the analogs of the target compound. The immunoassay established based on this kind of monoclonal antibody cannot be interfered by the other compounds and can detect the target compound accurately. In this study, an ic-ELISA analysis was established based on the prepared monoclonal antibody. As shown in Table 4, the cross-reaction rates for the GA analogs (syringic acid, protocatechuic acid, benzoic acid, caffeic acid, ferulic acid, and eugenol) were less than 0.09%. The result indicated that the method had excellent specificity for GA.

### 3.4. Analysis of Galla Chinensis by ic-ELISA

The average time to carry out the ic-ELISA analysis was 70 minutes. This method was high-throughput. In this view, it was time-saving compared with some other methods, e.g., HPLC and GC. The applicability of the present methodology to *G. chinensis* was evaluated, which also could display the accuracy and stability. The results of *G. chinensis* (GA content: 310.21 mg/g) diluted with different ratios are shown in Table 5. When the dilution ratios were 2 × 10^4^, 5 × 10^4^, and 10^5^, the resulting solutions were determined with the concentration of 1618.19 ± 46.07, 677.76 ± 26.32, and 327.63 ± 17.76 ng/mL, respectively. And the calculated content of gallic acid in *G. chinensis* was 323.64 ± 9.21, 338.88 ± 13.16, and 327.63 ± 17.76 mg/g, respectively. The coefficient of variation ranged from 2.85% to 5.42%. The results indicated that the ic-ELISA analysis exhibiting good accuracy and stability.

## 4. Conclusions

In this study, we designed and synthesized antigens with and without connecting arms and found that the introduction of connecting arms was beneficial for immune response. A specific mAb anti-GA-M702 was obtained by immunizing mice with GA-NH_2_-BSA. Based on this mAb, the ic-ELISA assay for GA detection was developed. It demonstrated good sensitivity and specificity. The method displayed good applicability for the determination of GA in *Galla chinensis*. In summary, the developed ic-ELISA assay provides an alternative approach to the rapid detection of GA in products.

## Figures and Tables

**Table 1:** Detection methods for GA.

Detection Methods	cLOD (mg/L)	Linear Range (mg/L)	Ref.
HPLC	0.03	0.26–5.18	[13]
GC	0.0025	0.50–5.00	[14]
DNP	0.051	0.17–8.51	[4]
EC	0.152	1.02–111.94	[15]
Solid-phase Extraction HPLC (SPE-HPLC)	4.66	4.66–233.00	[16]
FTIR	11.89	/	[17]
Capillary Electrophoresis (CE)	1.00	3.00–100.00	[18]

**Table 2:** Determination of serum titer of mice after the fifth immunization with GA-NH2-BSA.

Dilution Factor			P/N Value		
	MSZ 1-1	MSZ 1-2	MSZ 1-3	MSZ 1-4	MSZ 1-5
1/1k	59.20	78.88	76.04	72.12	92.48
1/3k	18.88	34.31	25.15	13.77	17.81
1/9k	7.43	11.79	7.89	5.50	4.96
1/27k	2.64	3.59	2.32	2.09	1.86
1/81k	1.62	1.86	1.48	1.33	1.24
1/243k	1.23	1.07	0.77	1.23	0.73
1/729k	0.21	0.17	0.00	0.42	0.21

**Table 3:** Determination of serum titer of mice after the fifth immunization with GA-BSA.

Dilution Factor			P/N Value		
	MSZ 2-1	MSZ 2-2	MSZ 2-3	MSZ 2-4	MSZ 2-5
1/1k	1.76	1.60	1.48	1.56	1.08
1/3k	1.15	1.27	1.12	1.08	1.04
1/9k	1.53	1.26	1.33	1.45	1.04
1/27k	0.77	0.85	0.73	0.69	0.65
1/81k	0.50	0.39	0.36	0.29	0.25
1/243k	0.08	0.13	0.17	0.13	0.17
1/729k	0.07	0.12	0.15	0.11	0.14

**Table 4:** Cross-reaction rates for the GA analogs.

GA Analogs	Cross Reaction Rate (%)
Gallic acid	100
Syringic acid	<0.09
Protocatechuic acid	<0.09
Benzoic acid	<0.09
Caffeic acid	<0.09
Ferulic acid	<0.09
Eugenol	<0.09
Gallicin	<0.09

**Table 5:** Analysis of GA in *G. chinensis* by ic-ELISA.

Dilution Factor	Solution Concentration (ng/mL)	Solution Detection Value (ng/mL)	Sample Content (mg/g)	Coefficient of Variation (%)
2 × 10^4^	1551.05	1618 ± 46	324 ± 9	2.85
5 × 10^4^	620.42	678 ± 26	339 ± 13	3.88
10^5^	310.21	328 ± 18	328 ± 18	5.42

## Data Availability

This article is a free-access publication.

## Supplementary Materials

The following supporting information can be downloaded at: https://www.mdpi.com/article/10.3390/bios14040182/s1, Table S1: Antibody titer detection; Table S2: Antibody subtype detection; Table S3: Determination of the optimum concentration of coating antigen; Table S4: Determination of the optimum concentration of mAb.
