# Supplementary material for: Development of a Monoclonal Antibody-Based Indirect Competitive Enzyme-Linked Immunosorbent Assay for the Rapid Detection of Gallic Acid
Source: Biosensors (Basel). 2024 Apr 9;14(4):182. doi: 10.3390/bios14040182 (PMC11048004; doi:10.3390/bios14040182)
Supplement: Supplementary file 1 [file biosensors-14-00182-s001.zip › biosensors-2929509-supplementary.pdf]

biosensors

MDPI

Supplemntary Materials

Development of a Monoclonal Antibody-Based Indirect Competitive Enzyme-Linked Immunosorbent Assay for the Rapid Detection of Gallic Acid

Jiajing Duan †, Xiuxia Zheng †, Ran Tao, Long Li, Fengzhong Wang, Yufeng Sun * and Bei Fan

Institute of Food Science and Technology, Chinese Academy of Agricultural Sciences, Beijing 100193, China; duanjiajing2021@163.com (J.D.); 18646654981@163.com (X.Z.); ran.tao5@mail.mcgill.ca (R.T.); llzgnydx@163.com (L.L.); wangfengzhong@sina.com (F.W.); fanbei@caas.cn (B.F.)

* Correspondence: yufengsuncaas@163.com

† These authors contributed equally to this work.

Table S1. Antibody potency detection.

Dilution factor	P/N value
500	42.17
1,500	20.90
4,500	8.83
13,500	4.21
40,500	2.12
121,500	1.33
364,500	0.87

Table S2. Antibody subtype detection.

Antibody subtype	IgG1	IgG2a	IgG2b	IgG3	IgM	IgA
OD_{450}	1.423	1.100	2.343	0.789	0.946	0.498

Table S3. Determination of the optimum concentration of coating antigen.

Coating antigen concentration（μg/mL）	A_{450}
1	1.146
0.5	1.043
0.25	0.951
0.125	0.734
0.0625	0.649
0.03125	0.580

Table S4. Determination of the optimum concentration of mAb.

mAb concentration（μg/mL）	Maximum competitive inhibition rate
2	1.29
0.67	1.29
0.22	0.91
0.074	0.75
0.025	0.62
